# Associations between psychotropic drugs and rsEEG connectivity and network characteristics: a cross-sectional study in hospital-admitted psychiatric patients

**DOI:** 10.3389/fnins.2023.1176825

**Published:** 2023-09-15

**Authors:** Melissa G. Zandstra, Hannah Meijs, Metten Somers, Cornelis J. Stam, Bieke de Wilde, Jan van Hecke, Peter Niemegeers, Jurjen J. Luykx, Edwin van Dellen

**Affiliations:** ^1^Department of Psychiatry, UMC Utrecht Brain Center, University Medical Center Utrecht, Utrecht University, Utrecht, Netherlands; ^2^Research Institute Brainclinics, Brainclinics Foundation, Nijmegen, Netherlands; ^3^Department of Cognitive Neuroscience, Faculty of Psychology and Neuroscience, Maastricht University, Maastricht, Netherlands; ^4^Department of Clinical Neurophysiology and MEG Center, Amsterdam UMC Location Vrije Universiteit Amsterdam, Amsterdam, Netherlands; ^5^Department of Psychiatry, Ziekenhuis Netwerk Antwerpen (ZNA), Antwerp, Belgium; ^6^Department of Neurology, Universitair Ziekenhuis (UZ), Brussels, Belgium; ^7^Vrije Universiteit Brussel, Brussels, Belgium

**Keywords:** electroencephalogram (EEG), psychotropic drugs, multi-class polypharmacy, functional connectivity, network organization, antipsychotics, antidepressants, benzodiazepines

## Abstract

**Introduction:**

Resting-state EEG (rsEEG) characteristics, such as functional connectivity and network topology, are studied as potential biomarkers in psychiatric research. However, the presence of psychopharmacological treatment in study participants poses a potential confounding factor in biomarker research. To address this concern, our study aims to explore the impact of both single and multi-class psychotropic treatments on aforementioned rsEEG characteristics in a psychiatric population.

**Methods:**

RsEEG was analyzed in a real-world cross-sectional sample of 900 hospital-admitted psychiatric patients. Patients were clustered into eight psychopharmacological groups: unmedicated, single-class treatment with antipsychotics (AP), antidepressants (AD) or benzodiazepines (BDZ), and multi-class combinations of these treatments. To assess the associations between psychotropic treatments and the macroscale rsEEG characteristics mentioned above, we employed a general linear model with *post-hoc* tests. Additionally, Spearman’s rank correlation analyses were performed to explore potential dosage effects.

**Results:**

Compared to unmedicated patients, single-class use of AD was associated with lower functional connectivity in the delta band, while AP was associated with lower functional connectivity in both the delta and alpha bands. Single-class use of BDZ was associated with widespread rsEEG differences, including lower functional connectivity across frequency bands and a different network topology within the beta band relative to unmedicated patients. All of the multi-class groups showed associations with functional connectivity or topology measures, but effects were most pronounced for concomitant use of all three classes of psychotropics. Differences were not only observed in comparison with unmedicated patients, but were also evident in comparisons between single-class, multi-class, and single/multi-class groups. Importantly, multi-class associations with rsEEG characteristics were found even in the absence of single-class associations, suggesting potential cumulative or interaction effects of different classes of psychotropics. Dosage correlations were only found for antipsychotics.

**Conclusion:**

Our exploratory, cross-sectional study suggests small but significant associations between single and multi-class use of antidepressants, antipsychotics and benzodiazepines and macroscale rsEEG functional connectivity and network topology characteristics. These findings highlight the importance of considering the effects of specific psychotropics, as well as their interactions, when investigating rsEEG biomarkers in a medicated psychiatric population.

## 1. Introduction

Precision psychiatry aims to shift current “one-size-fits-all” approaches based on categorical classifications, to a more targeted and individualistic approach (Fernandes et al., 2017). The key goal of this field is to develop tools that integrate an individual’s clinical and biological characteristics in order to more objectively inform clinical issues such as diagnosis, prognosis and treatment. One promising technique that could aid in this is quantitative electroencephalography (qEEG, Olbrich et al., 2015; Dharmadhikari et al., 2018; Widge et al., 2019). The technique is appealing due to its ability to analyze the brain at its core functional level, its broad availability and its cost-effectiveness.

EEG is typically comprised of delta (1–4 Hz), theta (4–8 Hz), alpha (8–12 Hz), beta (12–30 Hz), and gamma (>30 Hz) frequency bands (Canolty and Knight, 2010). Various psychiatric disorders have been associated with specific alterations in these frequency bands at rest (resting-state EEG or rsEEG). For instance, schizophrenia has been associated with consistent and reliable increases in slow frequency bands (delta and theta) and decreases in the alpha band, while depressive disorders are mainly associated with increased theta and beta power (Newson et al., 2019).

However, a critical consideration in rsEEG biomarker research is the potential confounding effects of medication commonly used to treat those disorders. Psychotropic medications, including antidepressants, antipsychotics, and benzodiazepines, exert diverse pharmacological actions that directly or indirectly modulate neurotransmitter systems. These actions can influence the excitability of neurons, synaptic connectivity, and overall brain function, thereby impacting EEG readings. This concern has been recognized since the early days of EEG research when Hans Berger discovered in 1933 that substances like barbiturates, scopolamine, and morphine could affect EEG readings (Berger, 1933).

Confounding effects of psychotropic agents on rsEEG readings hampers its application in clinical practice. For instance, in the context of clinical diagnosis, it becomes crucial to distinguish whether observed alterations in EEG patterns are primarily driven by the underlying psychiatric disorder or whether they can be attributed to the confounding effects of psychotropic medication. Moreover, it is important to disentangle the effects of the medication of interest from those of concomitant psychotropics, as combination therapy is common in psychiatric treatment. Therefore, a comprehensive understanding of the effects of different psychotropic agents on rsEEG is necessary to ensure accurate interpretation and clinical applicability of EEG findings.

Research on the effects of psychotropic drugs on rsEEG has primarily concentrated on power spectral measures, revealing changes induced by antidepressants (Knott et al., 2000; Siepmann et al., 2003; Saletu et al., 2006), benzodiazepines (Porjesz et al., 2002; Stone et al., 2015), and antipsychotics (Hyun et al., 2011). However, in the past two decades, more advanced techniques in EEG analysis, such as functional connectivity and network analysis, have emerged. These approaches offer potential as prediction tools (Rolle et al., 2020; Zhang et al., 2021). Despite the promise of this type of EEG analysis, the effects of psychotropic drugs on these metrics remain largely unexplored. The limited studies that have investigated the impact of psychotropics on these metrics (Knott et al., 2002; Leuchter et al., 2008; Numan et al., 2019) have predominantly focused on healthy populations and were based on small sample sizes. Moreover, these studies primarily examined monotherapy effects, which may not accurately reflect the complexities of real-world clinical settings where patients often receive single or multi-class polytherapy (Kukreja et al., 2013).

Given the limited existing literature on the effects of psychotropics on rsEEG functional connectivity and network topology metrics, our aim was to contribute valuable insights to this field of research. In this exploratory study, we examined the associations between commonly prescribed psychotropics, including antidepressants, antipsychotics and benzodiazepines, and aforementioned rsEEG metrics in a large naturalistic, cross-sectional population of hospital-admitted psychiatric patients. Importantly, our study sought to provide a comprehensive analysis by exploring not only the effects of single-class psychotropic treatments but also the potential interaction effects resulting from multi-class polypharmacy, providing a more realistic reflection of real-world treatment scenarios. Finally, associations were further examined by performing follow-up analyses to investigate dosage correlations. By shedding light on the potential confounding effects of single-class and multi-class treatments, our study could offer valuable insights that can enhance the accuracy and applicability of rsEEG biomarker research in clinical practice.

## 2. Materials and methods

### 2.1. Participants

Data were obtained between 2013 and 2018 at the Department of Psychiatry of the Ziekenhuis Netwerk Antwerpen (ZNA), a large community hospital in Antwerp, Belgium. Data collection procedures and study population characteristics were outlined previously (Kool et al., 2022). In brief, data were collected from 1,132 hospital-admitted adult patients that were diagnosed with at least one of the following psychiatric clinician-informed disorders based on DSM-IV: mood disorders (MD, depressive and bipolar disorders), psychotic disorders (PD, schizophrenia and other psychotic disorders, except brief psychotic disorder, and psychotic disorder due to a medical condition or substance use) and substance use disorders (SUD, alcohol and/or drug use disorders). Diagnoses were established using the clinical diagnostic interview by the clinician, a valid assessment and comparable to well-established standardized clinical interviews (Drill et al., 2015). Patients with two or three of the beforementioned disorders were assigned to a separate group called multiple morbidities (MM). The exclusion criteria were limited in order to gather a representative sample of patients with the abovementioned psychiatric disorders. Participants were excluded if they were unable to give informed consent, had restlessness that would affect EEG results, had poor quality EEG, or were using drugs falling outside the classes of antipsychotics (APs), antidepressants (ADs), and benzodiazepines (BDZs). All participants provided written informed consent. The institutional Review Board of the ZNA approved the study procedures that were carried out in accordance with the provisions of the World Medical Association Declaration of Helsinki.

### 2.2. EEG recordings

RsEEG recordings were made using a 64-channel Electrical Geodesics Incorporated (EGI) system (Philips, United States), at a sample frequency of 500 Hz. Participants underwent a three-minute eyes-closed rsEEG recording in a quiet (<40 dB) room. All participants were instructed to sit still and avoid excessive thinking to prevent resting-state mind wandering. The eyes closed condition was prefered over of the eyes open condition, because EEG parameters are more stable over sessions and the more prominent alpha oscillations in this condition provide good guidance for the selection of epochs (van Diessen et al., 2015). Raw EEG data were converted into ASCII files.

### 2.3. EEG preprocessing

Subsequent offline analysis was done in BrainWave version 0.9.152.12.26, developed by CJ Stam; further information and free software is available at: https://home.kpn.nl/stam7883/brainwave.html. EEG signals were filtered with a band-pass of 0.5-45 Hz. EEG data were visually inspected for artifacts by two independent raters. Artifacts included cardiac pulse, muscle tension, eye flutter or blink, and slow eye movement. Four channels associated with eye movements and one channel associated with a variety of other artifacts in a substantial number of participants were excluded from analysis (Supplementary Figure 1). Artifacts were allowed in a maximum of 6 channels (~10% of the remaining 59 channels), otherwise subjects were excluded from further analysis. The first 10 artifact-free epochs of 8.192 s each (~1.20 min in total) were selected. Reported outcome measures are based on averaged values over these 10 epochs per subject to further increase parameter stability (van Diessen et al., 2015; Colclough et al., 2016; Fraschini et al., 2016).

### 2.4. EEG data analysis

Data was re-referenced toward an average reference and filtered in four frequency bands, delta (0.5–4 Hz), theta (4–8 Hz), alpha (8–13 Hz) and beta (13–30 Hz). The gamma band (30–48 Hz) was excluded from analysis based on evidence suggesting that this frequency range cannot reliably be distinguished from muscle artifacts (Whitham et al., 2007). Macroscale rsEEG variables of interest included: amplitude envelope correlation corrected (AECc), phase lag index (PLI), and minimum spanning tree (MST) based on both the AECc and PLI.

#### 2.4.1. Functional connectivity

Functional connectivity can be based on amplitude and phase synchronization, which provide complementary information on connectivity between brain regions (Siems and Siegel, 2020). Two commonly used metrics that are not affected by volume conduction problems are the corrected amplitude envelope correlation (AECc) and phase lag index (PLI) (van Diessen et al., 2015; Colclough et al., 2016). By concurrently evaluating the AECc and PLI, our analysis offers a comprehensive understanding of the impacts of psychotropic substances on diverse aspects of functional connectivity.

##### 2.4.1.1. Phase lag index

The Phase lag index (PLI) measures the asymmetry of the distribution of phase differences between time series (Stam et al., 2007). The PLI ignores zero and π phase differences (Stam et al., 2007). Consequently, the method is less affected by the presence of common sources (volume conduction) than most other frequently used functional connectivity measures (Porz et al., 2014). The PLI is calculated by means of:


$$PLI=\;|\;<\mathrm{sign}\left[\sin\left(\Delta \phi \left(tk\right)\right)\right]\;>\;|\;\left(1\right)$$

Where sign is the signum function, ΔΦ is the phase difference between two time series (computed via the Hilbert transform), which is determined for all time-points (k) per epoch. <> indicates the mean value and || indicates the absolute value. The PLI value ranges from 0 to 1. A value of 0 indicates either a lack of coupling or coupling with a phase difference centered around 0 (mod π). A value of 1 means perfect phase locking. An extensive description of the PLI and its mathematical theory is provided by Stam et al. (2007).

##### 2.4.1.2. Amplitude envelope correlation corrected

The Amplitude Envelope Correlation (AEC) measures the synchrony between the oscillations of two time-series computing the correlation between their amplitude envelopes (Bruns et al., 2000). The AEC also ranges from 0 to 1 and high AEC values reflect synchronization between oscillations or networks. However, signal components that pick up the same source at different sites show an identical phase (Hipp et al., 2012). Therefore, we used the corrected version of the AEC (AECc), in which the time-series are orthogonalized by applying linear regression analysis (Hipp et al., 2012).

#### 2.4.2. Network organization

**Figure 1:**
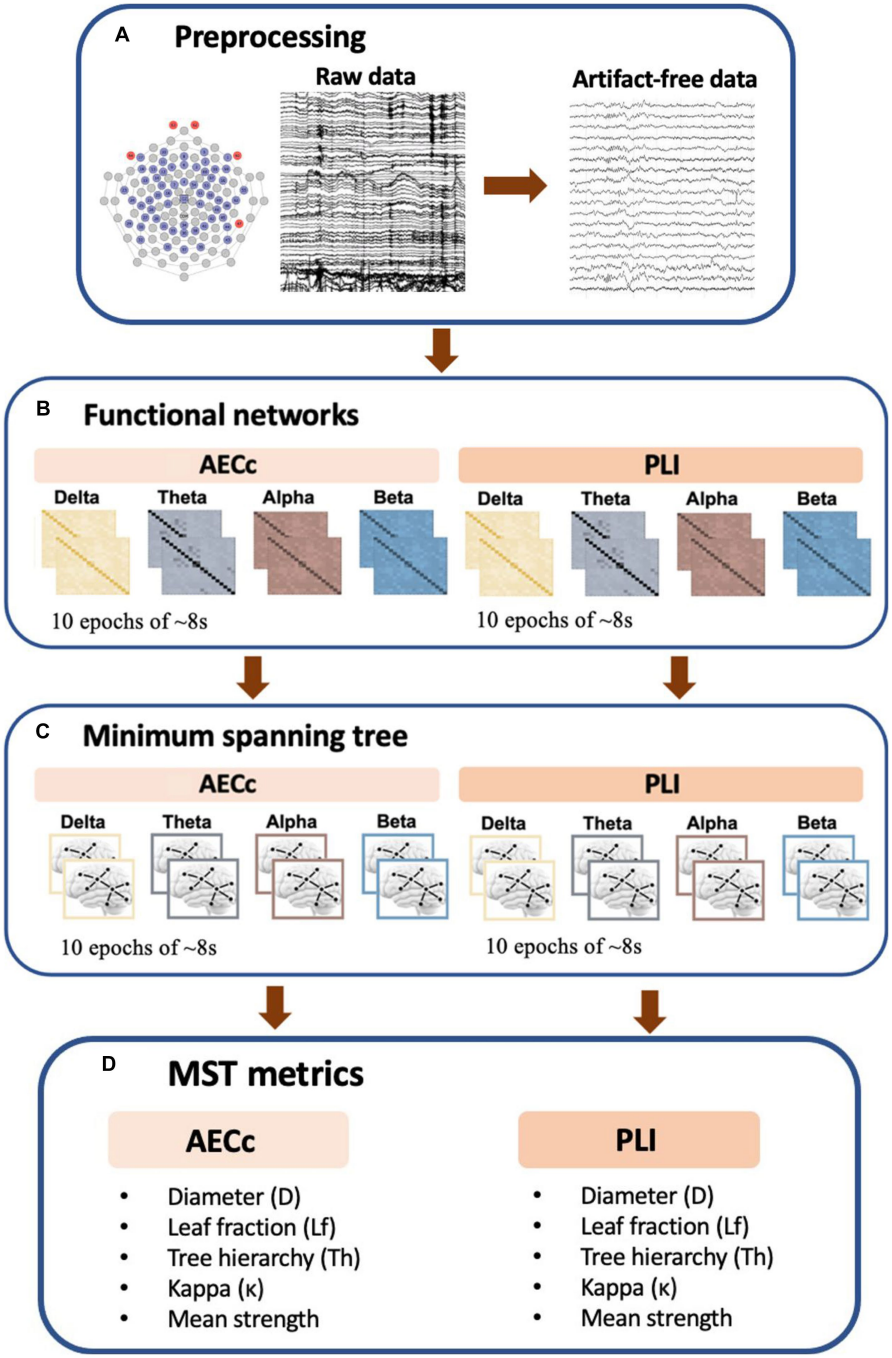
Schematic overview of EEG processing pipeline. **(A)** Raw rsEEG data was collected by a 64-channel EGI system. A band-pass filter was applied and epochs were visually inspected for artifacts. The first 10 artifact free epochs were selected for further analysis. **(B)** Functional connectivity matrices based on both the phase lag index (PLI) and amplitude envelope correlation corrected (AECc) were constructed for each frequency band (delta: 0.5–4 Hz, theta: 4–8 Hz, alpha: 8–13 Hz, and beta: 13–30 Hz) and each epoch. **(C)** Kruskal’s algorithm is applied to obtain the minimum spanning tree (MST) matrix. **(D)** To describe the topological properties of the tree, we used multiple metrics including the diameter (D), tree hierarchy (Th), leaf fraction (Lf), Kappa (κ), and mean strength.

Frequency-dependent brain network topology was assessed by constructing a minimum spanning tree (MST, Stam et al., 2014; Tewarie et al., 2015). An MST is the “backbone” of functional network organization, as it represents a unique acyclic subgraph of the strongest connections of the original weighted network (which are based upon functional connectivity measures) while minimizing the link weights (Stam et al., 2014; Tewarie et al., 2015). Furthermore, it is an unbiased method for comparing brain networks across conditions and gives information about the efficiency and integration of the network topology (Tewarie et al., 2015; van Diessen et al., 2015). The MST was calculated based on both the AECc and PLI connectivity matrices and consisted of 59 nodes (the number of nodes equaled the number of electrodes) and 58 edges (the number of edges was the number of nodes-1). There are a number of MST metrics that are used to describe the topological properties of the tree (Stam et al., 2014). We examined the following metrics; diameter (D), leaf fraction (Lf), kappa (κ), tree hierarchy (Th), and mean strength. The first four are network topology measures, but the last metric measures the functional connectivity strength within the aforementioned subgraph of the original weighted network and is therefore technically a functional connectivity measure. A detailed description of the beforementioned MST metrics can be found in Table 1. For a schematic overview of the EEG processing pipeline see Figure 1.

**Table 1:** Minimum spanning tree (MST) measures and their definitions.

MST metric	Explanation
Diameter (D)	Measure for network efficiency, where a low diameter indicates a more efficient the network. It is defined by the largest distance between any two nodes, normalized for the total number of connections.
Leaf fraction (Lf)	Measure for network integration, the higher (or closer to 1), the more integrated the network is. It is defined by the leaf number (number of nodes that have only one connection), divided by maximum possible leaf number.
Kappa (κ)	Measure of the width of the degree distribution (degree divergence) and relates to spread of information across the tree. High kappa indicates the presence of high-degree nodes which facilitate synchronization of the tree but also increase the network’s vulnerability if a hub is damaged.
Tree hierarchy (Th)	Measure that defines the hierarchy of the MST organization as optimal topology. It quantifies the trade-off between large scale integration in the MST and the overload of central nodes.
Strength	Mean weight of all edges included in the MST. Measures functional connectivity strength within the subgraph of the original weighted graph.

### 2.5. Drug treatment

#### 2.5.1. Psychotropic drug classes

Our analysis involved subjects who were treated with ADs, APs, BDZs, or combinations thereof. Multi-class combinations included: AP + BDZ, AD+BDZ, AP + AD, and AD+AP + BDZ. It is important to highlight that single-class treatments encompassed both monotherapy and single-class polytherapy, meaning that patients were allowed to receive two or more drugs from the same class (e.g., two different APs). We also included patients that were unmedicated. It is important to note that being unmedicated did not necessarily mean they had no prior history of medication, but rather they were not taking any medication at the time of data collection. For an overview of the specific drugs included in each class, see Table 2.

**Table 2:** Overview of specific drugs included within each class.

Name of class	Drugs included
AD	Amitriptyline, Buproprion, Citalopram, Clomipramine, Duloxetine, Dosulepine, Fluoxetine, Fluvoxamine, Imipramine, Melitracen, Mianserine, Mirtazapine, Nortriptyline, Paroxetine, Sertraline, Trazadone, Venlafaxine
AP	Amisulpride, Aripiprazole, Bromperidol, Clotiapine, Clozapine, Flupenthixol, Haloperidol, Olanzapine, Paliperidone, Pimozide, Pipamperone, Quetiapine, Risperidone, Sulpiride, Tiapride, Zuclopenthixol
BDZ	Alprazolam, Bromazepam, Chlorazepam, Clobazam, Clonazepam, Diazepam, Flunitrazepam, Flurazepam, Lorazepam, Lormetazepam, Nordazepam, Prazepam Zolpidem

AD, antidepressants; AP, antipsychotics; BDZ, benzodiazepines.

#### 2.5.2. Medication dose equivalence calculation methodology

Dose equivalents were calculated using the DDD (defined daily dose) methodology, endorsed by the WHO (WHO Collaborating Centre for Drug and Statistics Methodology, 2018) and recommended as the international standard for drug utilization monitoring and research. The DDD is defined as follows: the assumed average maintenance dose per day for a drug used for its main indication in adults (WHO Collaborating Centre for Drug and Statistics Methodology, 2018). DDDs are only assigned for drugs given an ATC (anatomical therapeutic chemical) code. The main advantage of this method is that DDDs are available for almost all psychotropic medications. Equivalents to diazepam for benzodiazepines, olanzapine for antipsychotics, and fluoxetine for antidepressants were calculated, using the ATC/DDD index of the WHO.

### 2.6. Statistical analysis

All statistical analyses were performed using IBM Statistics SPSS version 27 and ggplot2 (Wickham and Wickham, 2016) was used to generate plots. Parametric and, where appropriate, non-parametric tests were used.

Since specific psychotropic drugs may be prescribed for specific psychiatric disorders, a relationship between psychotropic treatment and diagnosis might exist within our data. In order to accommodate potential diagnosis-specific interaction effects, we first ran a multivariate general linear model (GLM) with diagnosis included as a fixed factor. Covariates included sex and age. If no diagnosis interaction was found, analyses were performed on all psychiatric disorders combined, otherwise separately.

After, we performed a second GLM with additional *post-hoc* tests with psychopharmacological group as fixed factor and sex and age as covariates. The psychopharmacological group omprised eight distinct categories: (1) no medication, (2) AD, (3) AP, (4) BDZ, (5) AD+AP, (6) AD+BDZ, (7) AP + BDZ, and (8) AP + AD+BDZ. A total of 48 rsEEG measures were included as outcome variables, including two connectivity measures (PLI and AECc) in four frequency bands, and five times two (both PLI and AECc) MST measures (D, Lf, Th, κ, strength) in four frequency bands. Frequency bands were: delta, theta, alpha, beta. In case of a diagnostic-specific interaction, we performed the abovementioned GLM four times (for PD, MD, SUD, and MM separately). To understand the dominant effect of medication on rsEEG characteristics in a real-world sample, the value of p significance threshold (α) of 0.05 was Bonferroni-corrected to control for type-I errors due to multiple testing (48 tests). While psychotropic medication effects on EEG connectivity and network organization is in itself a topic of interest, it may also be considered a confounder in pathophysiological and biomarker studies in psychiatric disorders. We therefore also performed analyses uncorrected for multiple testing to explore the potential confounding influence of psychotropic medication in such studies due to psychotropic medication. Both corrected and uncorrected significant findings were further examined by carrying out univariate one-way ANCOVAs and *post-hoc* tests (α = 0.05). Significant *post-hoc* tests were followed up by Spearman’s rank correlation analyses to investigate dosage-correlations. Dose equivalents were calculated using the defined daily dose methodology (WHO Collaborating Centre for Drug and Statistics Methodology, 2018).

## 3. Results

### 3.1. Demographic characteristics and medication use

Of 1,132 participants, 900 (406 females, mean age 41.4 ± 12.9 years) remained after EEG preprocessing and further data selection (for a flowchart of the data selection process: see Supplementary Figure 2). An overview of the demographics per psychopharmacological group can be found in Table 3. For a complete overview of the specific drugs included within each of the eight psychopharmacological groups, see Supplementary Table 1.

**Table 3:** Demographics per psychotropic group.

Demographic characteristic	No medication N = 190	AD N = 190	AP N = 65	BDZ N = 41	AD + AP N = 94	AD + BDZ N = 120	AP + BDZ N = 96	AD + AP + BDZ N = 104
Sex, female (%)	64 (33.7%)	92 (48.4%)	25 (38.5%)	23 (56.1%)	43 (45.7%)	66 (55%)	32 (33.3%)	61 (58.7%)
Age, mean (SD)	39.1 (12.8)	41.2 (12.8)	37.7 (13.3)	40.7 (15.1)	41.0 (12.4)	44.7 (13.3)	40.4 (12.9)	43.9 (12.7)
*Diagnosis*								
PD, N (%)	1 (0.5%)	3 (1.6%)	33 (50.8%)	3 (7.3%)	5 (5.3%)	5 (4.2%)	73 (76%)	31 (29.8%)
MD, N (%)	80 (42.1%)	88 (64.3%)		26 (63.4%)	39 (41.5%)	81 (67.5%)	8 (8.3%)	43 (41.4%)
SUD, N (%)	99 (51.1%)	79 (41.6%)	13 (20%)	10 (24.4%)	45 (47.9%)	26 (21.7%)	6 (6.3%)	15 (14.4%)
MM, N (%)	10 (5.3%)	20 (10.5%)	6 (9.2%)	2 (4.9%)	5 (5.3%)	8 (6.7%)	9 (9.4%)	15 (14.4%)
*Drug family* ^a^	NA			NA				
SSRI, N (%)		90 (47.4%)	NA		39 (41.5%)	61 (50.8%)	NA	55 (52.9%)
SNRI, N (%)		113 (59.5%)	NA		61 (64.9%)	63 (52.5%)	NA	57 (54.8%)
NDRI, N (%)		6 (3.2%)	NA		7 (7.5%)	3 (2.5%)	NA	5 (4.8%)
TCA, N (%)		34 (17.9%)	NA		15 (16%)	23 (19.2%)	NA	22 (21.1%)
FG, N (%)		NA	8 (12.3%)		19 (20.2%)	NA	8 (8.3%)	16 (15.8%)
SG, N (%)		NA	61 (93.9%)		84 (89.4%)	NA	119 (124%)	110 (105.7%)
*Dose range*	NA							
Olanzapine dose EQ (mg)		NA	0.5–45	NA	0.5–62	NA	0.6–75	0.3–60
Fluoxetine dose EQ (mg)		3.3–70	NA	NA	3.3–80	2.7–75	NA	2.7–100
Diazepam dose EQ (mg)		NA	NA	1.3–80	NA	0.3–60	0.9–60	03.90
*Mean dose* (SD)	NA							
Olanzapine dose EQ (mg)		NA	13.6 (11)	NA	7.5 (8.6)	NA	19.9 (14.7)	13.2 (13.6)
Fluoxetine dose EQ (mg)		22.6 (14.1)	NA	NA	26 (16.2)	24.7 (14.9)	NA	28.1 (16.9)
Diazepam dose EQ (mg)		NA	NA	19.3 (14.8)	NA	14 (9.9)	23 (13.8)	19.1 (14.2)

AD, antidepressants; AP, antipsychotics; BNZ, benzodiazepines; FG, first generation; EQ, equivalent; MD, mood disorder; MM, multiple morbidities; NDRI, norepinephrine dopamine reuptake inhibitor; PD, psychotic disorder; SG, second generation; SNRI, selective norepinephrine reuptake inhibitors; SSRI, selective serotonin reuptake inhibitors; SUD, substance use disorder; TCA, tricyclic antidepressants.

a Drug family exceeds total number of subjects, because most subjects were receiving polypharmacy: even within single-class treatment groups, subjects were commonly treated with more than one different kind of drug.

### 3.2. Interaction between treatment group and psychiatric disorder

In order to accommodate potential diagnosis-specific interaction effects, we first ran a GLM with psychopharmacological group and diagnosis as fixed factors and age and sex as covariates. The analysis yielded no significant interaction between diagnosis and psychopharmacological treatment, Wilks’ Lambda = 0.347, *F*(1,008, 15903.80) = 0.891, *p* = 0.993. Since there was no interaction, we performed analyses on all psychiatric disorders combined. The final GLM included psychopharmacological group as fixed factor and age and sex as covariates. This model yielded a significant main effect for psychopharmacological group, Wilks’ Lambda = 0.633, *F*(336, 5858.63) = 1.189, *p* = 0.012. Significant between-subjects effects and *post-hoc* results will be explained in detail in the following paragraphs.

### 3.3. Associations between psychotropic drugs and functional connectivity

A GLM was conducted in order to investigate associations between single and multi-class use of psychotropics and functional connectivity and network organization measures. For readability purposes, significant functional connectivity (including MST strength) results will be reported in this paragraph, while significant findings concerning network topology will be presented in a separate paragraph.

**Figure 2:**
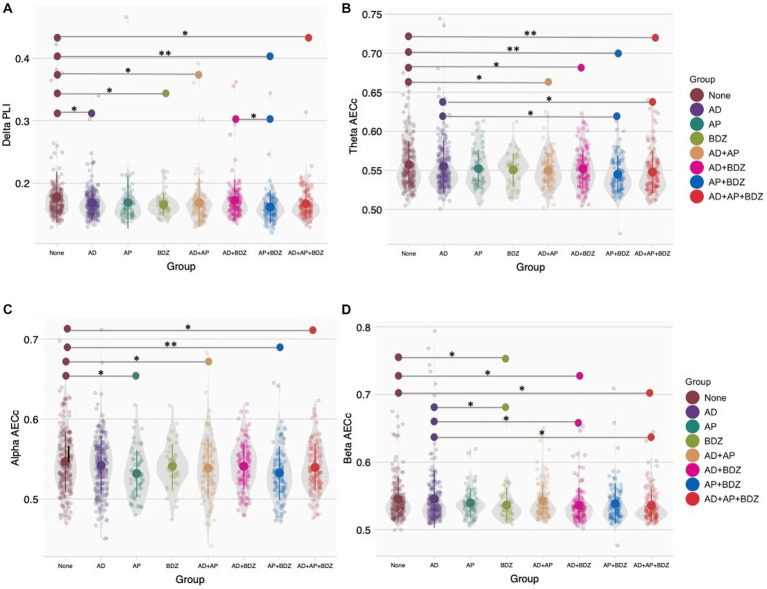
Group comparisons of functional connectivity **(A)** Phase lag index in the delta band, **(B)** Corrected amplitude envelope correlation in the theta band, **(C)** Corrected amplitude envelope correlation in the alpha band, **(D)** Corrected amplitude envelope correlation in the beta band. Group means and standard deviations are shown for no medication (brown), antidepressants (purple), antipsychotics (dark green), benzodiazepines (light green), antidepressant and antipsychotic polypharmacy (orange), antidepressant and benzodiazepine polypharmacy (pink), antipsychotic and benzodiazepine polypharmacy (blue), and polypharmacy of all three classes (red). The gray colored areas represent the kernel density estimation to show the distribution shape of the data. Little dots represent individuals. AD, antidepressants; AECc, amplitude envelope correlation corrected; AP, antipsychotics; BDZ, benzodiazepines; PLI, phase lag index. * Significant at an α-level of 0.05. ** Significant at an α-level of 0.01.

For functional connectivity measures, we found no significant group effects after Bonferroni correction (α = 0.05/48). Significant group effects uncorrected for multiple testing (α = 0.05) were found for the AECc in the theta band (*p* = 0.004, n_p_^2^ = 0.023), alpha band (*p* = 0.018, n_p_^2^ = 0.019), beta band (*p* = 0.044, n_p_^2^ = 0.016), and PLI in the delta band (*p* = 0.009, n_p_^2^ = 0.021). Significant GLM results and *post-hoc* results of between-group effects are shown in Table 4 (for an overview of all main results, as well as means and standard deviations of significant rsEEG findings: Supplementary Tables 2, 3). *Post-hoc* comparisons (α = 0.05) for functional connectivity measures revealed lower delta PLI in the BDZ, AD, AD+AP, AP + BDZ, and AD+AP + BDZ groups compared to untreated patients, as well as in AP + BDZ relative to AD+BDZ (Figure 2A). In addition, lower theta AECc was found in all multi-class groups compared to untreated patients and in the AP + BDZ and AD+AP + BDZ groups compared to AD (Figure 2B). The AECc within the alpha band was lower in the AP group and all multi-class groups containing antipsychotics compared to untreated patients (Figure 2C). Finally, compared to no medication and AD, we found lower beta AECc for BDZ, AD + BDZ, and AD+AP + BDZ (Figure 2D).

**Table 4:** Significant differences in functional connectivity metrics.

Functional connectivity measure	GLM results^a^	*Post-hoc* results^b^^,^^c^	
	Overall model	Group comparisons	
PLI delta band	*F* = 2.70	BDZ vs. None	*p* = 0.041 [−0.023–0.001]
	*p* = 0.009	AD vs. None	*p* = 0.009 [−0.016–0.002]
	n_p_^2^ = 0.021	AD+AP vs. None AP + BDZ vs. None AP + BDZ vs. AD+BDZ AD+AP + BDZ vs. None	*p* = 0.036 [−0.017–0.001] *p* = 0.00012 [−0.024–0.008] *p* = 0.017 [−0.020–0.002] *p* = 0.009 [−0.019–0.003]
AECc theta band	*F* = 3.04	AD+AP vs. None	*p* = 0.015 [−0.015–0.002]
	*p* = 0.004	AD+BDZ vs. None AP + BDZ vs. None	*p* = 0.034 [−0.013–0.001] *p* = 0.00032 [−0.019–0.006]
	n_p_^2^ = 0.023	AP + BDZ vs. AD AD+AP + BDZ vs. None AD+AP + BDZ vs. AD	*p* = 0.007 [−0.016–0.003] *p* = 0.0008 [−0.019–0.005] *p* = 0.015 [−0.015–0.002]
AECc alpha band	*F* = 2.44	AP vs. None	*p* = 0.002 [−0.025–0.005]
	*p* = 0.018	AD+AP vs. None AP + BDZ vs. None	*p* = 0.036 [−0.017–0.001] *p* = 0.0008 [−0.023–0.006]
	n_p_^2^ = 0.019	AD+AP + BDZ vs. None	*p* = 0.028 [−0.017–0.001]
AECc beta band	*F* = 2.70	BDZ vs. None	*p* = 0.029 [−0.023–0.001]
	*p* = 0.009	BDZ vs. AD AD+BDZ vs. None	*p* = 0.030 [−0.023–0.001] *p* = 0.003 [−0.019–0.004]
	n_p_^2^ = 0.021	AD+BDZ vs. AD AD+AP + BDZ vs. None AD+AP + BDZ vs. AD	*p* = 0.003 [−0.018–0.004] *p* = 0.006 [−0.020–0.003] *p* = 0.005 [−0.019–0.003]
PLI MST strength delta band	*F* = 3.73	BDZ vs. None	*p* = 0.037 [−0.040–0.001]
	***p* = 0.00054**	AP vs. None AD+AP vs. None	*p* = 0.016 [−0.034–0.004] *p* = 0.006 [−0.034–0.006]
	n_p_^2^ = 0.029	AP + BDZ vs. None AP + BDZ vs. AD AP + BDZ vs. AD+BDZ AP + BDZ vs. AD+AP + BDZ AD+AP + BDZ vs. None	*p* = 0.000007 [−0.047–0.019] *p* = 0.004 [−0.035–0.007] *p* = 0.0008 [−0.042–0.11] *p* = 0.045 [−0.033–0.001] *p* = 0.024 [−0.030–0.002]
AECc MST strength theta band	*F* = 2.86	BDZ vs. None	*p* = 0.039 [−0.037–0.001]
	*p* = 0.006	AD+AP vs. None AP + BDZ vs. None	*p* = 0.007 [−0.023–0.004] *p* = 0.0005 [−0.027–0.007]
	n_p_^2^ = 0.022	AP + BDZ vs. AD AD+AP + BDZ vs. None	*p* = 0.012 [−0.022–0.003] *p* = 0.003 [−0.024–0.005]
AECc MST strength alpha band	*F* = 3.95	BDZ vs. None	*p* = 0.019 [−0.029–0.003]
	***p* = 0.00030**	AP vs. None AD+AP vs. None	*p* = 0.002 [−0.028–0.006] *p* = 0.007 [−0.023–0.004]
	n_p_^2^ = 0.030	AP + BDZ vs. None AP + BDZ vs. AD AD+AP + BDZ vs. None	*p* = 0.000016 [−0.030–0.011] *p* = 0.007 [−0.023–0.004] *p* = 0.0006 [−0.026–0.007]
PLI MST strength alpha band	*F* = 2.28	AD+BDZ vs. AP	*p* = 0.039 [−0.070–0.002]
	*p* = 0.027	AD+AP + BDZ vs. None AD+AP + BDZ vs. AD	*p* = 0.006 [−0.066–0.011] *p* = 0.010 [−0.062–0.008]
	n_p_^2^ = 0.018	AD+AP + BDZ vs. AP AD+AP + BDZ vs. AD+AP	*p* = 0.006 [−0.084–0.014] *p* = 0.007 [−0.075–0.012]

η_p_^2^, partial Eta squared; AECc, amplitude envelope correlation corrected; MST, minimum spanning tree; PLI, phase lag index.

a Results of multivariate GLM and *post-hoc* tests. Only GLM results for which significant results were found are displayed (see Supplementary material for all results). Covariates in the model: age, sex. Note that *P*-values reported here are uncorrected; GLM statistical significance was based on Bonferroni-corrected (α = 0.05/48) and uncorrected (α = 0.05) significance thresholds for multiple testing. Significant GLM findings meeting the Bonferroni-corrected threshold (α = 0.05/48) are depicted in bold.

b *Post-hoc* tests were uncorrected (α = 0.05) for multiple testing. Only significant post-hoc results are mentioned.

c 99% confidence intervals are reported between brackets.

**Figure 3:**
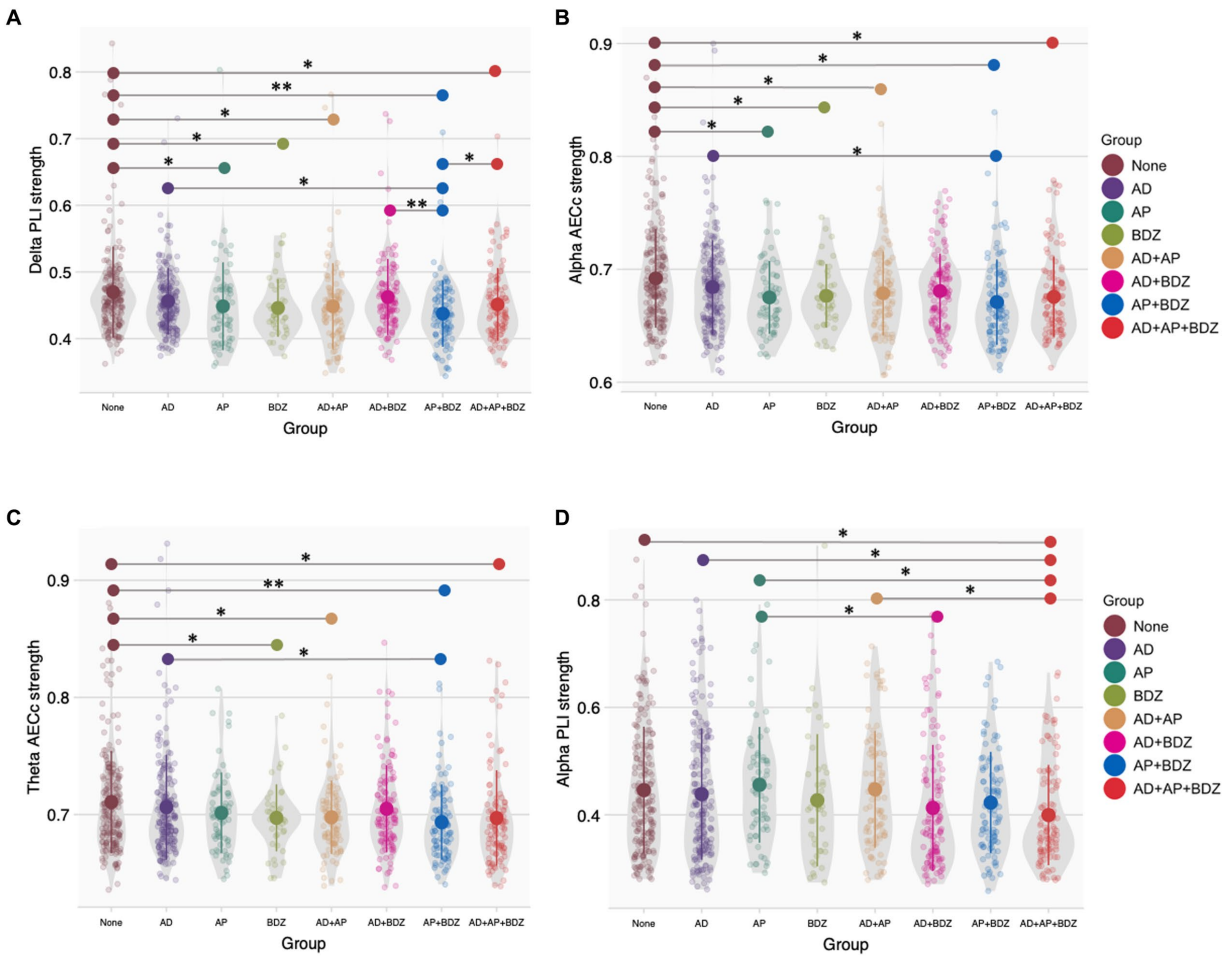
Group comparisons of functional connectivity (MST) strength **(A)** Phase lag index strength in the delta band, **(B)** Corrected amplitude envelope correlation strength in the alpha band, **(C)** Corrected amplitude envelope correlation strength in the theta band, **(D)** Phase lag index strength in the alpha band. Group means and standard deviations are shown for no medication (brown), antidepressants (purple), antipsychotics (dark green), benzodiazepines (light green), antidepressant and antipsychotic polypharmacy (orange), antidepressant and benzodiazepine polypharmacy (pink), antipsychotic and benzodiazepine polypharmacy (blue), and polypharmacy of all three classes (red). The gray colored areas represent the kernel density estimation to show the distribution shape of the data. Little dots represent individuals. AD, antidepressants; AECc, amplitude envelope correlation corrected; AP, antipsychotics; BDZ, benzodiazepines; PLI, phase lag index. * Significant at an α-level of 0.05. ** Significant at an α-level of 0.01.

For functional connectivity (MST) strength measures, we found Bonferroni-corrected group effects for PLI strength in the delta band (*p* < 0.001, n_p_^2^ = 0.029) and AECc strength in the alpha band (*p* < 0.001, n_p_^2^ = 0.030). We found lower delta PLI strength in all groups (except AD and AD+BDZ) compared to untreated patients, as well as in AP + BDZ compared to both AD, AD+BDZ, and AD+AP + BDZ (Figure 3A). In addition, lower alpha AECc strength was found in all groups (except AD and AD+BDZ) compared to untreated patients, as well as in AP + BDZ compared to AD (Figure 3B). Uncorrected group effects were found for AECc strength in the theta band (*p* = 006, n_p_^2^ = 0.022) and PLI strength in the alpha band (*p* = 027, n_p_^2^ = 0.018). *Post-hoc* tests revealed lower theta AECc strength for BDZ and all multi-class groups containing antipsychotics compared to untreated patients, as well as in the AP + BDZ group relative to AD (Figure 3C). Finally, the alpha PLI strength was found to be lower for AD+AP + BDZ compared to untreated patients, AP, AD, and AD+AP, as well as in AD+BDZ compared to AP (Figure 3D).

#### 3.3.1. Dosage correlations with functional connectivity measures

Spearman’s rank correlation tests were conducted between the dose of the psychotropic of interest and the aforementioned significant functional connectivity (strength) outcomes within the group that showed significance. Within the AD+AP group, we found a significant negative correlation between antipsychotic dose and delta PLI (*r* = −0.309, *p* = 0.002) and delta PLI strength (*r* =  −0.229, *p* = 0.026). Within the AD+AP + BDZ group, we found significant negative correlations between antipsychotic dose and theta AECc (*r* =  −0.243, *p* = 0.013) and alpha AECc (*r* =  −0.210, *p* = 0.032). No other correlations yielded statistical significance.

### 3.4. Associations between psychotropic drugs and network organization

For network organization measures, we found no significant group effects after Bonferroni correction (α = 0.05/48). Group effects (significant when uncorrected for multiple testing) were found for a variety of network characteristics in the delta, alpha and beta bands. More specifically, we found effects for the PLI-κ within the delta (*p* = 033, n_p_^2^ = 0.017) and alpha (*p* = 032, n_p_^2^ = 0.017) bands, the AECc-Th tree hierarchy in the alpha band (*p* = 046, n_p_^2^ = 0.016) and AECc-D diameter in the beta band (*p* = 046, n_p_^2^ = 0.016). Significant GLM results and *post-hoc* results of between-group effects are shown in Table 5 (for an overview of all main results, as well as means and standard deviations of significant rsEEG findings: Supplementary Tables 2, 3).

**Table 5:** Significant differences in network topology metrics.

Network organization measure	GLM results^a^	*Post-hoc* results^b^^,^^c^	
	*Overall model*	*Group comparisons*	
PLI-κ delta band	*F =* 2.19	AD+BDZ vs. BDZ	*p* = 0.019 [−0.598–0.053]
	*p =* 0.033	AD+AP vs. AD+BDZ	*p* = 0.010 [−0.473–0.064]
	*n_p_^2^ =* 0.017	AP + BDZ vs. None AP + BDZ vs. AD AP + BDZ vs. AD+BDZ	*p* = 0.048 [−0.372–0.001] *p* = 0.025 [−0.399–0.027] *p* = 0.0006 [−0.563–0.154]
PLI-κ alpha band	*F =* 2.20	AD+BDZ vs. None	*p* = 0.006 [−0.606–0.100]
	*p =* 0.032	AD+AP + BDZ vs. None AD+AP + BDZ vs. AD	*p* = 0.001 [−0.704–0.175] *p* = 0.015 [−0.585–0.062]
	*n_p_^2^ =* 0.017	AD+AP + BDZ vs. AP AD+AP + BDZ vs. AD+AP	*p* = 0.020 [−0.718–0.036] *p* = 0.044 [−0.619–0.008]
AECc-Th alpha band	*F =* 2.06	AD vs. AP BDZ vs. AP	*p* = 0.027 [−0.013–0.001] *p* = 0.012 [−0.020–0.002]
	*p =* 0.046	AD+AP + BDZ vs. None	*p* =0.017 [−0.012–0.001]
	*n_p_^2^* = 0.016	AD+AP + BDZ vs. AP AD+AP + BDZ vs. AP + BDZ	*p* = 0.001 [−0.019–0.005] *p* = 0.019 [−0.014–0.001]
AECc-D beta band	*F = 2.06*	BDZ vs. None	*p* = 0.005 [0.004–0.024]
	*p = 0.046*	BDZ vs. AD	*p* = 0.011 [0.003–0.022]
	*n_p_^2^ = 0.016*	BDZ vs. AD+AP AD+AP + BDZ vs. None	*p* = 0.032 [0.001–0.022] *p* = 0.025 [0.001–0.015]

η_p_^2^, partial Eta squared; AECc, amplitude envelope correlation corrected; D, diameter; κ, kappa, PLI, phase lag index; Th, tree hierarchy.

a Results of multivariate GLM and *post-hoc* tests. Only GLM results for which significant results were found are displayed (see Supplementary material for all results). Covariates in the model: age, sex. Note that *P*-values reported here are uncorrected; GLM statistical significance was based on Bonferroni-corrected (α = 0.05/48) and uncorrected (α = 0.05) significance thresholds for multiple testing. Significant GLM findings meeting the Bonferroni-corrected threshold (α = 0.05/48) are depicted in bold.

b *Post-hoc* tests were uncorrected (α = 0.05) for multiple testing. Only significant post-hoc results are mentioned.

c 99% confidence intervals are reported between brackets.

**Figure 4:**
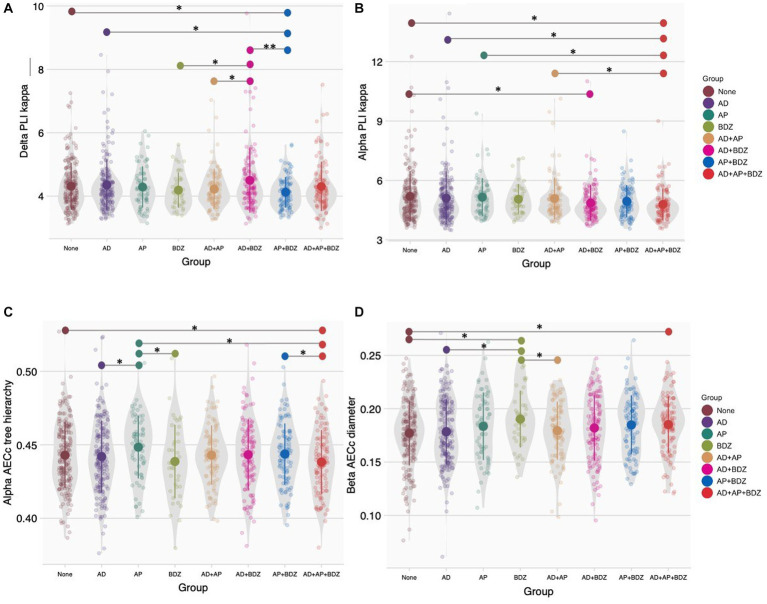
Group comparisons of network topology **(A)** Phase lag index kappa in the delta band, **(B)** Phase lag index kappa in the alpha band, **(C)** Corrected amplitude envelope correlation tree hierarchy in the alpha band, **(D)** Corrected amplitude envelope correlation diameter in the alpha band. Group means and standard deviations are shown for no medication (brown), antidepressants (purple), antipsychotics (dark green), benzodiazepines (light green), antidepressant and antipsychotic polypharmacy (orange), antidepressant and benzodiazepine polypharmacy (pink), antipsychotic and benzodiazepine polypharmacy (blue), and polypharmacy of all three classes (red). The gray colored areas represent the kernel density estimation to show the distribution shape of the data. Little dots represent individuals. AD, antidepressants; AECc, amplitude envelope correlation corrected; AP, antipsychotics; BDZ, benzodiazepines; PLI, phase lag index. * Significant at an α-level of 0.05. ** Significant at an α-level of 0.01.

*Post-hoc* tests (α = 0.05) found the delta PLI-κ to be lower in AP + BDZ compared to untreated patients, AD and AD+BDZ, as well as in AD+BDZ relative to BDZ and AD+AP (Figure 4A). Within the alpha band, we found lower PLI-κ in the AD+AP + BDZ group compared to untreated patients, AD, AP, and AD+AP. In addition, we found this metric to be lower in de AD+BDZ group compared to untreated patients (Figure 4B). Furthermore, we found lower AECc-Th in AD+AP + BDZ relative to unmedicated patients, AP and AP + BDZ, as well as in AD and BDZ compared to AP (Figure 4C). Finally, higher AECc-D was found for the BDZ group compared to untreated patients, AD and AD+AP, as well as in AD+AP + BDZ relative to untreated patients (Figure 4D).

#### 3.4.1. Dosage correlations with network topology measures

Spearman’s rank correlation tests were conducted between the dose of the psychotropic of interest and the aforementioned significant network topology outcomes within the groups that showed statistical significance. Within the AD+AP group we found a significant negative correlation between antipsychotic dose and delta PLI-k (*r* = −0.279, *p* = 0.006). No other correlations yielded statistical significance.

## 4. Discussion

In a retrospective cross-sectional study, we explored associations between single and multi-class psychotropic treatment and macroscale rsEEG functional connectivity and topology measures in a hospital-admitted psychiatric population. The classes of psychotropic drugs investigated were antipsychotics, antidepressants, and benzodiazepines. Bonferroni-corrected main effects were only found for functional connectivity (MST) strength in the delta (PLI), and alpha band (AECc). Both measures were lower in the antipsychotic and benzodiazepine single-class groups and the majority of multi-class groups compared to unmedicated patients. The absence of additional significant corrected effects could potentially be attributed to variations in treatment duration, dosage, underlying disease etiology, disorder severity, or use of a stringent correction method. Exploratory analyses, which were uncorrected for multiple testing, suggest effects of both single and multi-class use of psychotropics on a range of connectivity and topology measures. Differences were found both in comparison with untreated patients and between different psychopharmacological groups. Notably, we only found dosage correlations for antipsychotics. Although effect sizes were too small to withstand Bonferroni correction, our findings may still have implications for EEG biomarker research.

### 4.1. Single-class psychotropic associations with EEG connectivity and network topology

Associations between single-class use of antidepressants and rsEEG metrics were only found for the functional connectivity (PLI) strength in the delta band, which was lower in this group compared to unmedicated patients. We are not aware of previous studies on the effects of antidepressants on macroscale connectivity and topology measures. However, previous studies have reported regional connectivity decreases in the alpha band of depressed men (Iseger et al., 2017) and in the theta band of healthy men (Nissen et al., 2020), using linear lagged connectivity (LLC) and PLI, respectively. It is possible that our macroscale approach may have obscured these regional differences. Additionally, the discrepancies between our findings and the aforementioned studies may be attributed to variations in study design, study population characteristics, and gender composition. When considering the study by Iseger and colleagues, the inconsistencies between their findings and our own could also be potentially elucidated by the variance in the connectivity metric used. Both the LLC and PLI are measures of phase synchronization. However, the LLC is a measure that examines the linear relationship between two signals (Pascual-Marqui, 2007), while the PLI examines nonlinear relations (Stam et al., 2007). Consequently, it is plausible that antidepressants could impact linear connectivity within the alpha band while not significantly affecting nonlinear connectivity. We are unaware of studies examining the effects of antidepressants on linear and nonlinear connectivity. However, it is noteworthy to mention that a study involving benzodiazepines demonstrated contrasting impacts on linear and nonlinear connectivity (Alonso et al., 2010).

Antipsychotics were found to be associated with lower functional connectivity strength in the delta band (PLI) and alpha band (AECc and AECc-strength) compared to unmedicated patients. In contrast to our study, a recent systematic review revealed no systematic effects of antipsychotics on macroscale EEG connectivity and network characteristics (Mackintosh et al., 2021). Only one of the reviewed studies directly assessed the impact of antipsychotics on EEG connectivity by comparing pre-and post-antipsychotic drug treatment in treatment refractory psychosis patients using EEG intra-and interhemispheric correlation analysis (Cerdán et al., 2005). Interestingly, they observed regional connectivity increases across various frequencies, with the exception of a connectivity decrease in the high theta band. The discrepancies between our study and the aforementioned research may stem from differences in the study populations, brain regions analyzed (macroscale vs. regional), and the specific connectivity metrics employed. The metric used by Cerdán and colleagues is sensitive to volume conduction. Volume conduction can cause electrical or magnetic signals generated in one region to spread to neighboring areas (van den Broek et al., 1998), which can result in an erroneous estimate of the actual connectivity between brain areas. Therefore, the use of this metric has been discouraged (Colclough et al., 2016). Consequently, the reliability and validity of the outcomes from the study by Cerdán and colleagues might be somewhat compromised.

Associations between benzodiazepines and rsEEG metrics were more widespread. We found lower connectivity (strength) across frequency bands (i.e., delta band PLI and PLI-strength, theta and alpha AECc-strength, and beta AECc) in patients treated with benzodiazepines compared to unmedicated patients. In the beta band, we also found a higher diameter compared to unmedicated patients, single-class use of antidepressants, indicating less efficient information processing between remote brain regions (Tewarie et al., 2015). Furthermore, we identified network topology differences between single-class use of benzodiazepines and other psychopharmacological groups. For example; the diameter in the beta band was also higher compared to single-class use of antidepressants and multi-class use of antidepressants and antipsychotics, the AECc tree hierarchy in the alpha band was lower compared to single-class use of antipsychotics, and the PLI kappa in the delta band was lower compared to multi-class use of benzodiazepines and antidepressants. The majority of previous studies have primarily focused on investigating the effects of benzodiazepines on regional rsEEG metrics. However, there have been a limited number of studies that specifically explored the impact of benzodiazepines on macroscale connectivity metrics. In line with our findings, Arnts and colleagues observed a decrease in beta band functional connectivity (AECc) throughout the brain in patients with severe brain injury after administration of zolpidem (Arnts et al., 2020). Again, in line with our study, previous research using the PLI found no effect of benzodiazepines in the beta band in healthy controls (Alonso López et al., 2015) or patients scheduled for a gastrointestinal endoscopic procedure (Numan et al., 2019). These findings suggest that benzodiazepines affect beta band amplitude coupling but not phase-coupling.

### 4.2. Multi-class psychotropic associations with EEG connectivity and network topology

A complex pattern of multi-class psychotropic associations was found for both functional connectivity and network measures (AECc and PLI) across frequency bands. All of the multi-class groups showed associations with functional connectivity or network measures, but effects were most pronounced for patients treated with all three psychotropics and least pronounced in patients treated with antidepressants and benzodiazepines. Importantly, we found differences between patients treated with multi-class combinations of psychotropics and unmedicated patients even when no association was found when patients on single-class treatment with one of these agents were compared to unmedicated patients.

In case of both single and multi-class associations, the multi-class associations may be driven by the psychotropic class that also showed a single-class association. For example, the lower AECc in the beta band found in patients concomitantly treated with antidepressants and benzodiazepines relative to unmedicated patients was also found for single-class use of benzodiazepines but not for single-class use of antidepressants. In fact, both single-class benzodiazepine and multi-class antidepressant and benzodiazepine groups showed a lower AECc beta compared to single-class antidepressant group, which had the exact same mean value as unmedicated patients. Thus, although single-class use of a psychotropic (in this case antidepressants) may not affect rsEEG metrics, concomitant use with another psychotropic might actually influence rsEEG outcomes, meaning that multi-class effects may be entirely attributed to one class of psychotropics.

Multi-class associations with connectivity and network measures were also found while no association was observed when patients on single-class treatment with these agents were compared to unmedicated patients (e.g., AECc strength in the theta band). This suggests that combinations of psychotropics may have summative effects on EEG connectivity and topology. We speculate that the regional effects of antidepressants, antipsychotics and benzodiazepines described in the literature (see previous section) can result in macroscale functional connectivity and network alterations when multiple psychotropics are combined. Another explanation may be that complex interactions between drugs are responsible for the observed effects. For example, one study found that benzodiazepines reduce dopamine release by activation of GABAA receptors (Brodnik et al., 2018). This suggests that the interactions of benzodiazepines and antipsychotics may result in an even greater decrease in dopaminergic activity compared to using antipsychotic treatment alone treatment with antipsychotic treatment alone, which might consequently result in decreased connectivity. Many other possible interaction effects may exist, and the complex interactional effects of psychotropic agents on macroscale EEG characteristics are of interest for future studies.

### 4.3. Implications

The observed associations between both single-class and multi-class use of psychotropics and functional connectivity and network topology metrics were relatively small. However, even small associations can have important implications for EEG biomarker research. These associations may impact the ability to distinguish between medication and disease effects of used medication and the effects of the underlying condition being studied. In addition, it is important to disentangle the effects of the psychotropic of interest from concomitant psychotropics. Therefore, it is crucial to carefully consider and control the influence of psychotropic medications when interpreting EEG connectivity and topology findings. In particular, the use of benzodiazepines, and to a lesser extent, antidepressants and antipsychotics, should be recognized as potential confounding factors in studies examining EEG connectivity and topology. Notably, our study revealed that multi-class associations with macroscale connectivity and network organization measures may be present even in the absence of significant single-class psychotropic effects. This highlights the importance of considering the cumulative impact of multiple psychotropic medications when investigating EEG biomarkers. Furthermore, our findings indicate that the dose of antipsychotics may play a role, as we observed dose-effects in this particular medication class. This suggests that both the use of antipsychotics and the specific dosage should be taken into account as relevant covariates in rsEEG studies involving medicated psychiatric patients. For antidepressants and benzodiazepines, the agents may be important covariates regardless of the specific dosage. In summary, our study underscores the need to carefully consider and control the effects of psychotropic medications, especially benzodiazepines and multi-class combinations of psychotropics, in EEG connectivity and topology studies. Understanding and accounting for these medication-related factors will enhance the accuracy and reliability of EEG biomarker research in psychiatric populations.

### 4.4. Strengths and limitations

Strengths of our study include the large cross-diagnostic sample in a real-world setting of hospital-admitted psychiatric patients, analysis of a range of rsEEG connectivity and network topology parameters, and analysis of multiple psychotropics with a focus on both single-class and multi-class associations, reflecting clinical practice.

Several limitations should be borne in mind when interpreting the results, mostly stemming from its retrospective design. First, due to the cross-sectional study design, we were unable to compare EEG results before and after treatment. Our results therefore provide an estimation of between-subject effects of psychotropic medication, rather than within-subject effects. Second, it is important to acknowledge that our sample consisted of individuals from a psychiatric population. While we did not observe an interaction effect between diagnosis and psychopharmacological group, we cannot dismiss the possibility of disease-specific effects, such as the influence of disease etiology or symptoms, on the studied EEG metrics. Therefore, the observed differences in EEG patterns may be attributed to various factors related to the underlying psychiatric disorders rather than solely the effect of treatment. While spectral EEG studies have provided insights into disorder-related effects on rsEEG (Newson et al., 2019), investigations on the effects on connectivity and topology measures have been limited and require further research to elucidate their significance. Third, we could not control for several confounding factors, such as smoking and caffeine consumption immediately prior to obtaining EEG data, which are known factors to influence EEG results (Siepmann and Kirch, 2002; Fisher et al., 2012). In addition, the use of non-prescribed drugs or psychedelics has not been documented and therefore cannot be ruled out. However, it is worth noting that the use of psychedelics in Belgium during the time of data collection was very limited. According to a report from 2018, only 0.5% of the Flemish population between the ages of 15 and 64 reported using LSD or hallucinogenic mushrooms in the past 12 months (Drieskens et al., 2019). Fourth, it has been found that EEG measures are influenced by both short-term pharmacological effects of drugs and patients’ long-term clinical responses to medication (Saletu et al., 2006), however, we did not have information regarding treatment duration. Fifth, the retrospective nature of our study also limited our ability to capture detailed and standardized information on medication dosages. While dosing was accounted for within the available data, the lack of precise dosing control may have influenced the results. Future research directions should consider employing prospective study designs or complementary methods such as randomized controlled trials to achieve greater control over dosing. This would allow for a more in-depth analysis of the effects of different dosage levels on rsEEG connectivity and topology metrics. Sixth, subjects using multiple different medications within the same class (same-class polytherapy) made up the majority of the single-class groups, and due to heterogeneity in treatment regimes, we were not able to take specific combinations into account in our analyses. Drug interactions may not only exist between drugs of different classes, but also between drugs within the same class. Thus, our results may not be representative for patients under strict monotherapy. For antipsychotics, the receptor binding profiles of typical and atypical antipsychotics are quite different, while they both bind to the dopamine D_2_ receptor, the latter also binds to a variety of other receptors (Kusumi et al., 2015). RsEEG effects may be larger for atypical antipsychotics than for typical antipsychotics (Centorrino et al., 2002). In the present study, the majority of patients taking antipsychotics were on atypical agents. Similarly, the binding profiles of different subclasses of antidepressants vary (Sánchez and Hyttel, 1999), which may differentially affect rsEEG metrics. Therefore, future investigations should aim to explore the effects of different subclasses of psychotropic medications on rsEEG connectivity and topology. By addressing these factors, future research can provide a more comprehensive understanding of the relationship between psychotropic medications and rsEEG measures. Finally, although it is a strength that our study focused on both phase and amplitude synchronization, many different metrics exist within these two broad categories. Future research should focus on examining the effects of psychotropics on various metrics within these categories. For instance, the potential differential effects on linear and nonlinear metrics could be a fruitful avenue for future research.

## 5. Conclusion

Taken together, the present study provides evidence that, in a cross-sectional, hospital-admitted psychiatric population, small macroscale alterations of the strength and organization of functional connectivity were present in patients treated with single-class antidepressants, antipsychotics and benzodiazepines, as well as in multi-class combinations of these psychotropics. Whereas single-class use of antidepressants was specifically associated with functional connectivity of the delta band, we found more widespread associations for antipsychotics, benzodiazepines and multi-class combinations of psychotropics with beforementioned measures. Importantly, multi-class associations were also found in the absence of single-class associations, suggesting summative or interaction effects between different classes of psychotropics. Our study highlights the importance of considering the effects of specific psychotropics, as well as their interactions, when investigating rsEEG biomarkers in a medicated psychiatric population, ultimately leading to enhanced accuracy and applicability or rsEEG connectivity and topology metrics in clinical practice. Further research is needed to validate and expand upon our findings, and to explore the underlying mechanisms driving these associations.

## Data availability statement

The raw data supporting the conclusions of this article will be made available by the authors, without undue reservation.

## Ethics statement

The studies involving humans were approved by the institutional Review Board of the ZNA. The studies were conducted in accordance with the local legislation and institutional requirements. The participants provided their written informed consent to participate in this study.

## Author contributions

JL, BW, ED, and JH conceived the study. BW, JL, JH, and PN collected data. HM preprocessed data. HM and MZ conducted statistical analyses. MZ wrote the first draft. HM, CS, MS, JL, and ED reviewed the manuscript. MS, JL, and ED supervised the project. All authors contributed to the article and approved the submitted version.

## Funding

Funding for this project was obtained through two personal UMC Utrecht Brain Center Rudolf Magnus Young Talent Fellowships (H150) to JL and to ED, respectively; and by the Dutch Organization for Health Research and Development (ZonMW) under Grant Agreement No. 60-63600-98-711 (personal grant to ED).

## Conflict of interest

The authors declare that the research was conducted in the absence of any commercial or financial relationships that could be construed as a potential conflict of interest.

## Publisher’s note

All claims expressed in this article are solely those of the authors and do not necessarily represent those of their affiliated organizations, or those of the publisher, the editors and the reviewers. Any product that may be evaluated in this article, or claim that may be made by its manufacturer, is not guaranteed or endorsed by the publisher.
