# Supplementary material for: Associations between psychotropic drugs and rsEEG connectivity and network characteristics: a cross-sectional study in hospital-admitted psychiatric patients
Source: Front Neurosci. 2023 Sep 15;17:1176825. doi: 10.3389/fnins.2023.1176825 (PMC10541222; doi:10.3389/fnins.2023.1176825)
Supplement: Supplementary file 1 [file Data_Sheet_1.pdf]

Supplementary Material

Associations between psychotropic drugs and rsEEG connectivity and network characteristics: a cross-sectional study in hospital-admitted psychiatric patients

Melissa G. Zandstra[1*], Hannah Meijs[2,3], Metten Somers[1], Cornelis J. Stam[4], Bieke de Wilde[5], Jan van Hecke[5], Peter Niemegeers[5], Jurjen J. Luykx[1,2,3], Edwin van Dellen[1,6]

[1] Department of Psychiatry, UMC Utrecht Brain Center, University Medical Center Utrecht, Utrecht University, Utrecht, The Netherlands

[2] Research Institute Brainclinics, Brainclinics Foundation, Nijmegen, The Netherlands

[3] Department of Cognitive Neuroscience, Faculty of Psychology and Neuroscience, Maastricht University, Maastricht, The Netherlands

[4] Department of Clinical Neurophysiology and MEG Center, Amsterdam UMC location Vrije Universiteit Amsterdam, De Boelelaan 1117, Amsterdam, The Netherlands

[5] Department of Psychiatry, Ziekenhuis Netwerk Antwerpen (ZNA), Antwerp, Belgium

[6] Department of Neurology, Universitair Ziekenhuis (UZ) Brussel and Vrije Universiteit Brussel, Brussels, Belgium.

*** Correspondence:**
Melissa G. Zandstra, MD
m.g.zandstra@umcutrecht.nl

Tables

Supplementary Table 1. Overview of specific drugs included within each psychopharmacological group

	Antipsychotics		Antidepressants				Benzodiazepines
**Group (N)**	**FG, drug (N)**	**SG, drug (N)**	**SSRI, drug (N)**	**SNRI, drug (N)**	**TCA, drug (N)**	**NDRI, drug (N)**	**Drug (N)**
No medication (190)	NA	NA	NA	NA	NA	NA	NA
AD (190)	NA	NA	Escitalopram (43)<br>Sertraline (20)<br>Citalopram (10)<br>Fluoxetine (9)<br>Paroxetine (8)	Trazodone (76)<br>Venlafaxine (23)<br>Duloxine (14)	Mirtazapine (26)<br>Amitriptyline (5)<br>Clomipramine (3)	Bupropion (6)	NA
AP (65)	Pipamperon (4)<br>Haloperidol (3)<br>Zuclopentixol (1)	Olanzapine (31)<br>Quetiapine (16)<br>Aripiprazole (8)<br>Clozapine (2)<br>Clotiapine (1)<br>Risperidone (2)<br>Paliperidone (1)	NA	NA	NA	NA	NA

BNZ (41)	NA	NA	NA	NA	NA	NA	Lormetazepam (15)<br>Diazepam (9)<br>Lorazepam (8)<br>Zolpidem (8)<br>Alprazolam (1)<br>Clonazepam (1)<br>Flunitrazepam (1)<br>Clobazam (1)<br>Clonazepam (1)<br>Nordazepam (1)<br>Chloorazepam (1)
AD+AP (94)	Pipamperon (9)<br>Flupentixol (7)<br>Broomperidol (1)<br>Sulpiride (1)<br>Pimozide (1)	Quetiapine (34)<br>Olanzapine (20)<br>Rispiridone (11)<br>Clotiapine (7)<br>Aripiprazole (4)<br>Amisulpride (4)<br>Paliperidone (3)<br>Clozapine (1)	Escitalopram (15)<br>Sertraline (10)<br>Paroxetine (11)<br>Fluoxetine (3)	Trazodone (39)<br>Venlafaxine (16)<br>Duloxetine (6)	Melitracen (7)<br>Mirtazapine (3)<br>Amitriptyline (3)<br>Clomipramine (2)	Bupropion (7)	NA
AD+BNZ (120)	NA	NA	Escitalopram (29)	Trazodone (46)	Mirtazapine (12)	Bupropion (3)	Lormetazepam (25)<br>Alprazolam (25)

			Paroxetine (9)<br>Citalopram (8)<br>Sertraline (8)<br>Fluoxetine (6)<br>Fluvoxamine (1)	Venlafaxine (14)<br>Duloxetine (3)	Amitriptyline (6)<br>Dosulpine (2)<br>Clomipramine (1)<br>Mianserine (1)<br>Nortriptyline (1)		Diazepam (21)<br>Zolpidem (19)<br>Lorazepam (17)<br>Flurazepam (5)<br>Chloorazepam (2)<br>Prazepam (2)<br>Bromazepam (2)
AP+BNZ (96)	Broomperidol (3)<br>Haloperidol (2)<br>Flupentixol (1)<br>Pimozide (1)<br>Pipamperone (1)	Olanzapine (54)<br>Quetiapine (21)<br>Clotiapine (13)<br>Aripirazole (13)<br>Paliperidone (8)<br>Rispiridone (5)<br>Amisulpride (4)<br>Clozapine (1)	NA	NA	NA	NA	Lorazepam (55)<br>Lormetazepam (35)<br>Diazepam (14)<br>Alprazolam (8)<br>Clonazepam (3)<br>Chloorazepam (3)<br>Zolpidem (2)<br>Flurazepam (1)<br>Prazepam (1)
AD+AP+BNZ (104)	Flupentixol (10)<br>Sulpride (3)<br>Pipamperone (2)<br>Haloperidol (1)	Olanzapine (38)<br>Quetiapine (37)<br>Amisulpride (11)<br>Clotiapine (11)<br>Rispiridone (7)	Escitalopram (19)<br>Fluoxetine (11)<br>Paroxetine (11)<br>Sertraline (11)<br>Citalopram (3)	Trazodone (38)<br>Venlafaxine (16)<br>Duloxetine (3)	Clomipramine (6)<br>Melitracen (5)<br>Mirtazapine (4)<br>Nortriptyline (4)<br>Amitriptyline (3)	Bupropion (5)	Lorazepam (34)<br>Alprazolam (24)<br>Lormetazepam (23)<br>Diazepam (22)<br>Zolpidem (10)<br>Bromazepam (5)<br>Clonazepam (5)

	Aripiprazole (3)	Chloorazepam (3)
	Clozapine (3)	Flurazepam (3)
		Flunitrazepam (1)

Abbreviations: AD, antidepressants; AP, antipsychotics; BDZ, benzodiazepines; FG, first generation; IA, intermediate acting; LA, long acting; SA, short acting; SG, second generation; SNRI, serotonin norepinephrine reuptake inhibitor; SSRI, selective serotonin reuptake inhibitor; TCA, tricyclic antidepressants.

Supplementary Table 2. Overview of GLM results

**rsEEG variables**		***F***	***p-value***[a]	**η_p^2**
AECc	*Delta*	1.574	.139	.012
	Theta	3.039	.004*	.023
	Alpha	2.442	.018*	.019
	Beta	2.074	.044*	.016
MST	*Delta-D*	.945	.471	.007
	Theta-D	1.629	.141	.012
	Alpha-D	.808	0.581	.006
	Beta-D	2.057	.046*	.016
	Delta- κ	.796	.590	.006
	Theta- κ	1.633	.122	.013
	Alpha- κ	1.613	.128	.013
	Beta- κ	1.650	.118	.013
	Delta-Lf	.419	.891	.003
	Theta-Lf	1.219	.289	.009
	Alpha-Lf	1.694	.107	.013
	Beta-Lf	1.306	.244	.010
	Delta-Th	.346	.933	.003
	Theta_Th	.788	.598	.006
	Alpha-Th	2.047	.046*	.016
	Beta-Th	.857	.541	.007
	Delta strength	1.946	.060	.015
	Theta strength	2.856	.006*	.022
	Alpha strength	3.945	.00005**	.030
	Beta strength	1.733	.098	.013
PLI	*Delta*	2.698	.009*	.021
	Theta	1.837	.077	.014
	Alpha	1.725	.100	.013
	Beta	1.204	.298	.006
MST	*Delta-D*	.424	.888	.003
	Theta-D	.869	.531	.007
	Alpha-D	.701	.671	.005
	Beta-D	.403	.901	.003
	Delta- κ	2.185	.033*	.017
	Theta- κ	.936	.477	.007
	Alpha- κ	2.202	.032*	.017
	Beta- κ	.923	.488	.007
	Delta-Lf	.727	.649	.006
	Theta-Lf	1.781	.088	.014
	Alpha-Lf	1.751	.094	.014
	Beta-Lf	.824	.567	.006
	Delta-Th	.625	.736	.005
	Theta_Th	.818	.572	.006
	Alpha-Th	.931	.482	.007
	Beta-Th	1.342	.227	.010
	Delta strength	3.733	.0005**	.029
	Theta strength	1.213	.293	.009
	Alpha strength	2.278	.027*	.018
	Beta strength	.913	.496	.007

Abbreviations: η_p^2 = partial Eta squared; κ, kappa; AECc, amplitude envelope correlation with leakage correction; D, diameter; Lf, leaf fraction; MST, minimum spanning tree; PLI, phase lag index; Th, tree hierarchy.

[a] Note that *p*-values are true values

[*] Statistically significant without correction ($\alpha = 0.05$).

[**] Statistically significant after Bonferroni correction ($\alpha = 0.05/48$). Significant *p*-values meeting this threshold were found for alpha AECc strength and delta PLI strength.

Supplementary Table 3. Overview of means and standard deviations of significant findings

rsEEG variable		No medication		AD		AP		BDZ		AP+AD		AD+BDZ		AP+BDZ		AD+AP+BDZ	
		**M (SD)**	**$M^{adjusted}$ (SD)**	**M (SD)**	**$M^{adjusted}$ (SD)**	**M (SD)**	**$M^{adjusted}$ (SD)**	**M (SD)**	**$M^{adjusted}$ (SD)**	**M (SD)**	**$M^{adjusted}$ (SD)**	**M (SD)**	**$M^{adjusted}$ (SD)**	**M (SD)**	**$M^{adjusted}$ (SD)**	**M (SD)**	**$M^{adjusted}$ (SD)**
AECc	*Theta*	.557 (.03)	.558 (.002)	.555 (.034)	.555 (.002)	.552 (.024)	.553 (.003)	.551 (.021)	.550 (.004)	.550 (.022)	.550 (.003)	.552 (.024)	.551 (.003)	.545 (.023)	.546 (.003)	.548 (.028)	.547 (.003)
	Theta strength	.712 (.044)	.711 (.003)	.706 (.045)	.706 (.003)	.701 (.035)	.702 (.005)	.697 (.029)	.697 (.006)	.698 (.032)	.698 (.004)	.705 (.037)	.705 (.004)	.694 (.032)	.694 (.004)	.697 (.041)	.697 (.004)
	Alpha	.547 (.037)	.548 (.002)	.542 (.036)	.542 (.002)	532 (.029)	.532 (.004)	.541 (.028)	.540 (.005)	.539 (037)	.539 (.003)	.541 (.029)	.540 (.003)	.532 (.033)	.533 (.003)	.539 (.029)	.538 (.003)
	Alpha strength	.692 (.043)	.692 (.003)	.684 (.040)	.684 (.003)	.675 (.032)	.675 (.005)	.676 (.028)	.676 (.006)	.679 (.037)	.679 (.004)	.681 (.033)	.681 (.004)	.671 (.038)	.671 (.004)	.676 (.036)	.676 (.004)
	Alpha-Th	.443 (.022)	.444 (.002)	.442 (.024)	.442 (.002)	.448 (.021)	.449 (.003)	.439 (.025)	.438 (.003)	.443 (.020)	.443 (.002)	.443 (.024)	.443 (.002)	.444 (.021)	.445 (.002)	.438 (.022)	.437 (.002)

	Beta	545	547	546	547	540	538	536	535	543	544	536	536	539	537	536	535
		(.034)	(.002)	(.043)	(.002)	(.022)	(.004)	(.026)	(.005)	(.028)	(.003)	(.026)	(.003)	(.030)	(.003)	(.026)	(.003)
	Beta-D	.177	.177	.178	.179	.184	.184	.190	.191	.179	.180	.182	.181	.185	.184	.185	.185
		(.030)	(.002)	(.031)	(.002)	(.032)	(.004)	(.027)	(.004)	(.027)	(.003)	(.031)	(.003)	(.028)	(.003)	(.026)	(.003)
PLI	*Delta*	.178	.177	.168	.169	.169	.169	.166	.166	.169	.169	.173	.172	.162	.162	.167	.167
		(.041)	(.002)	(.025)	(.002)	(.042)	(.004)	(.018)	(.005)	(.039)	(.003)	(.034)	(.003)	(.026)	(.003)	(.024)	(.003)
	Delta strength	.470	.469	.456	.457	.449	.449	.446	.448	.448	.449	463	463	.437	.436	.451	.452
		(.068)	(.004)	(.050)	(.004)	(.066)	(.007)	(.043)	(.009)	(.065)	(.006)	(.057)	(.005)	(.050)	(.006)	(.055)	(.006)
	Delta-K	4.312	4.322	4.365	4.349	4.289	4.288	4.189	4.173	4.227	4.225	4.498	4.494	4.121	4.135	4.305	4.294
		(.739)	(.055)	(.817)	(.055)	(.652)	(.094)	(.558)	(.118)	(.635)	(.078)	(1.02)	(.070)	(.484)	(.077)	(.780)	(.074)
	Alpha strength	.446	.442	.438	.439	.455	.453	.427	.429	.447	.447	.413	.417	.423	.420	.400	.404
		(.118)	(.008)	(.112)	(.008)	(.108)	(.014)	(.112)	(.018)	(.109)	(.012)	(.117)	(.010)	(.094)	(.011)	(.093)	(.011)
	Alpha-K	5.196	5.210	5.009	5.094	5.151	5.148	5.047	5.007	5.086	5.084	4.861	4.858	4.948	4.969	4.786	4.771
		(1.05)	(.080)	(1.34)	(.079)	(.970)	(.136)	(.774)	(.171)	(1.06)	(.112)	(.909)	(.101)	(.815)	(.111)	(.813)	(.108)

Abbreviations: AECc, amplitude envelope correlation with leakage correction; AD, antidepressants; AP, antipsychotics; BDZ, benzodiazepines; PLI, phase lag index.

Figures

Supplementary Figure 1. Layout of 64-channel EGI system

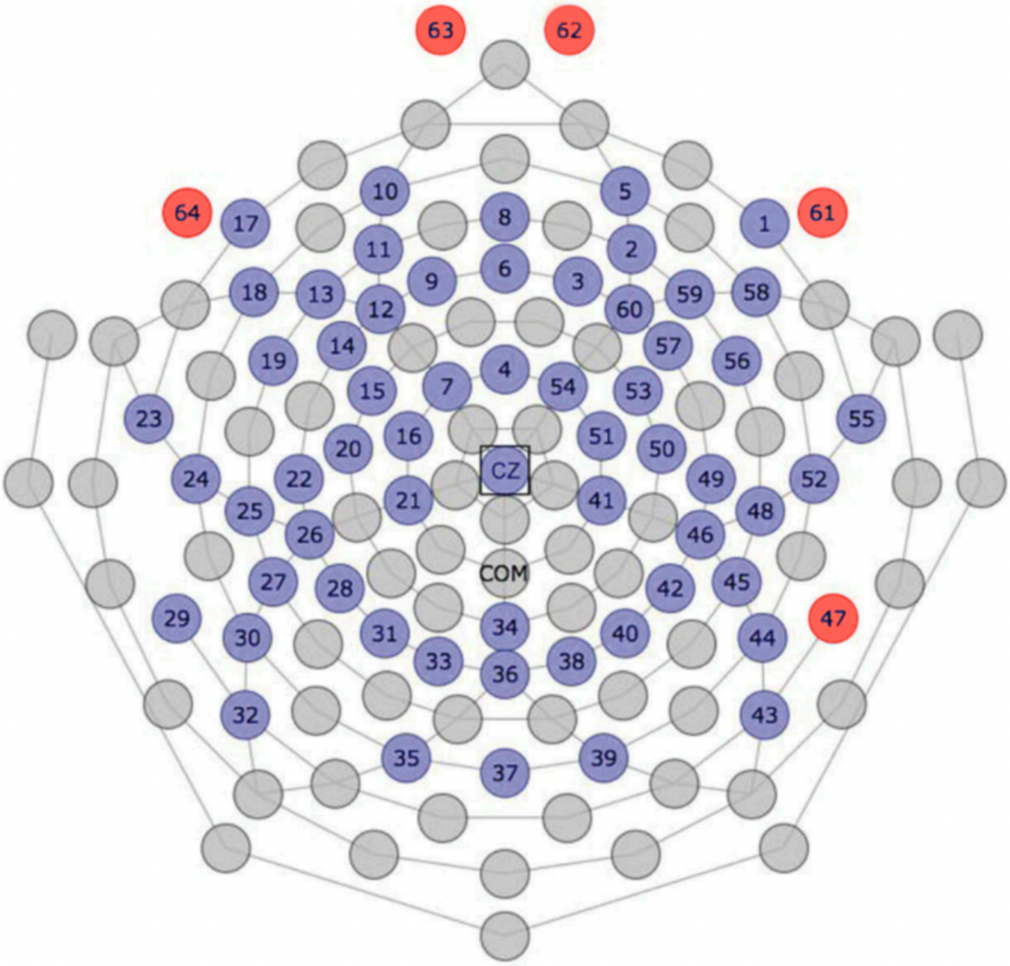

EEGs were recorded with 64 channels plus the reference channel (65, Cz) of the EEG Geodesic Sensor Net by Electrical Geodesics Incorporation (EGI). Electrodes depicted in purple represent electrodes used for analysis, whereas red circles depict electrodes that were excluded due to the presence of eye movements (61,62,63,64) or other artifacts (47) in a large number of participants.

Supplementary Figure 2. Flowchart of data selection process

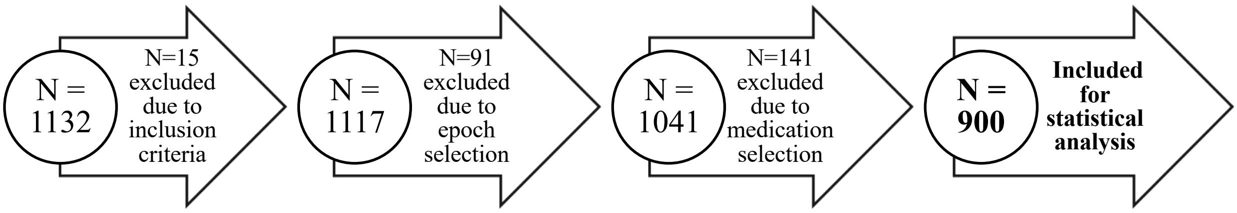

EEG data were available for 1132 potentially eligible individuals, who provided written informed consent. Of these subjects, 15 did not meet inclusion criteria. From the EEGs of the remaining 1117 subjects, the first 10 artifact-free epochs were selected. Subjects whose EEG recordings contained major artifacts in more than 10% of all electrodes, were excluded (N = 91). In this way, the epochs of remaining 1041 subjects were either artifact-free or contained artifacts in only a limited number of channels (up to 6 electrodes). Finally, we excluded subjects who used medications other than antidepressants, antipsychotics, or benzodiazepines, as well as subjects who used the aforementioned medications in combination with any other medication that did not belong to one of these three groups. This resulted in a total of 900 subjects that were included for statistical analysis.
